# Epidemiological profile of road traffic injury patients, and post-traumatic stress disorder (PTSD) screening uptake in hospitals in Fako Division, Cameroon

**DOI:** 10.1371/journal.pgph.0004847

**Published:** 2025-12-09

**Authors:** Claudia Ngeha Ngu, Priya Shete, Dickson Shey Nsagha, Elvis Asangbeng Tanue, Nicholas Tendongfor, Rasheedat Oke, Nahyeni Bassah, Chrisantus Eweh Ukah, Sandra I. McCoy, Catherine Juillard, Alain Chichom Mefire, Edie Gregory Halle Ekane

**Affiliations:** 1 Department of Public Health and Hygiene, Faculty of Health Sciences, University of Buea, Buea, Cameroon; 2 Department of Medicine, University of California San Francisco, San Francisco, California, United States of America; 3 Program for the Advancement of Surgical Equity, Department of Surgery, University of California Los Angeles, Los Angeles, California, United States of America; 4 Division of Epidemiology, School of Public Health, University of California Berkeley, Berkeley, California, United States of America; University of Chicago, UNITED STATES OF AMERICA

## Abstract

Road traffic injuries (RTIs) are a major global public health concern, often leading to serious physical and psychological consequences, including post-traumatic stress disorder (PTSD). A limited understanding of specific RTI patient characteristics, particularly regarding PTSD screening recommendation and uptake in health facilities in Cameroon, hinders the development of effective PTSD management strategies. This study aimed to determine the epidemiological profile of RTI patients and PTSD screening uptake in hospitals in Fako Division, Cameroon. A chart review of medical records of RTI patients received between 2019 and 2023 was conducted from July 30^th^, 2024 to August 30^th^, 2024 at three hospitals in Fako Division, Cameroon: Buea Regional Hospital, Saint Luke Hospital, and Limbe Regional Hospital. A data extraction form was used to collect demographic information, injury characteristics, and PTSD screening recommendations and uptake. We used Chi-square test to investigate associations between independent variables and PTSD screening recommendation and uptake. A multivariable logistic regression model was built to identify independent predictors. A total of 4218 RTI patients were included. Patients were predominantly male, 69.6% (2937/4218). The age group of 26–35 years recorded the highest proportion, 31.7% (1339/4218). Only 12.7%(534/4218) of patients were hospitalized, with only 11.0% (59/534) of hospitalized patients recommended for PTSD screening, and 44.1% (26/59) of those recommended undergoing screening. Independent predictors for PTSD screening recommendation included sedative given (AOR = 6.5, 95%CI = 1.4-30.1), psychotherapy recommended (AOR = 19.2, 95%CI = 8.9-41.6), and transfusion performed (AOR = 3.5, 95%CI = 1.5-7.9). PTSD screening uptake was significantly associated with recommendation for psychotherapy (COR = 4.2, 95%CI = 1.4-12.7). Males were mostly involved. PTSD screening recommendation (11.0%) and uptake (44.1%) were low among RTI patients. PTSD screening uptake was significantly associated with recommendation for psychotherapy. This underscores the urgent need for systematic PTSD screening and integrated mental health services to develop tailored management strategies and improve outcomes in Fako Division and similar contexts.

## Introduction

Road traffic injuries (RTIs) represent a major global public health challenge, causing over 1.19 million deaths and approximately 50 million injuries each year, with disproportionate consequences in low- and middle-income countries (LMICs) through higher rates of long-term disability and economic burden [1–3]. In Cameroon, estimates indicate 16,583 annual road crashes, resulting in over 6,000 deaths, and a loss of 1,443 disability‑adjusted life years (DALYs) per 100,000 population [1,4–6]. The increasing incidence of RTIs in Cameroon is attributed to several factors, including rapid urbanization, poor road infrastructure, limited enforcement of road‑safety regulations, and the increasing availability of motorcycles as an affordable means of transport [6,7,8]. These factors contribute to a higher risk of RTIs, particularly among vulnerable road users such as pedestrians and motorcyclists [7,9,10].

RTI can have severe psychological consequences, including post-traumatic stress disorder (PTSD) [11,12]. PTSD is a prevalent debilitating psychological disorder following RTIs, with a pooled global prevalence of 22.2%, and studies show that 1 in 4 RTI victims in Africa develop PTSD [13,14]. In the context of this study, psychotherapy refers to structured, evidence-based therapeutic interventions (e.g., Cognitive Behavioral Therapy, Exposure Therapy) delivered by trained mental health (MH) specialists (psychiatrists or psychologists) following a formal diagnosis of PTSD. While MH care has been incorporated into the primary healthcare in Cameroon [15,16], routine PTSD screening (commonly recommended at ≥1 month after RTI/ trauma, allowing resolution of acute stress disorder) is not part of standard RTI care [17]. Specialist mental health (MH) providers (psychiatrists, clinical psychologists) are scarce and concentrated in major urban centers [15,18–20]. As a result, task‑sharing approaches are used where general practitioners initiate screening and management recommendations (including psychotherapy), deliver supportive counseling, and refer complicated cases to specialists’ care. Formal diagnostic assessment and structured psychotherapy are typically provided by trained MH professionals where resources permit [15]. General practitioners may make initial recommendations for PTSD assessment and management or provide preliminary psychosocial support based on clinical judgment, guides, and observation of symptoms. However, the decision for structured psychotherapy and its delivery remains solely with trained MH specialists after a definitive diagnosis [15].

Despite the growing burden of RTIs and its link with PTSD, data on RTI patients’ epidemiological profiles and PTSD screening uptake (herein refers to the proportion of patients who undergo PTSD screening after recommendation) remain limited in Cameroon, particularly at the sub‑national level, as prior studies focused on demographics and injury patterns [10,21]. Injuries constitute a significant proportion of emergency visits in Cameroon and Fako Division in particular [22,23], yet data on PTSD screening uptake ≥ 1 month post‑RTI is rare, suggesting knowledge gaps. This study aimed to determine the epidemiological profile of RTI Patients and PTSD screening uptake in hospitals in Fako Division, Cameroon, with the goal of providing data to inform the development of integrated trauma-mental health services in resource‑limited settings based on local evidence.

## Methodology

### Ethics statement

This study was approved by the Institutional Review Board of the Faculty of Health Sciences of the University of Buea (No: 2024/2524-04/UB/SG/IRB/FHS) and administrative authorization from the South West Regional Delegation of Public Health. To maintain confidentiality since authors had access to information that could identify individual participants during data collection, a code was assigned to each reviewed RTI patient’s record for de-identification.

### Study design

This study employed a retrospective chart review of medical records of RTI patients received and managed between January 2019 and December 2023 at three purposefully selected hospitals in Fako Division, Cameroon: Buea Regional Hospital, Saint Luke Hospital Buea, and the Limbe Regional Hospital. The chart review was done from July 30^th^, 2024 to August 30^th^, 2024. These hospitals were selected based on their trauma care capabilities and geographical location, which enable them to receive a significant number of RTI victims.

### Study setting and population

**Fig 1:**
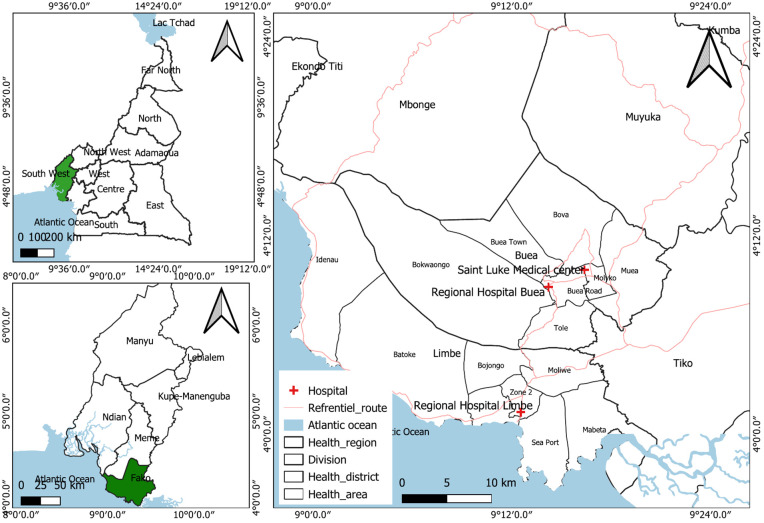
Map of three hospital sites, Fako Division, Cameroon. Base map produced using QGIS with shape files obtained from the Humanitarian Data Exchange in collaboration with OCHA Cameroon via the dataset “Cameroon - Subnational Administrative Boundaries” available at https://data.humdata.org/dataset/cod-ab-cmr. This data is licensed under Creative Commons Attribution for Intergovernmental Organisations (CC BY-IGO), which is compatible with the CC BY 4.0 license.

The study was conducted in Fako Division, Cameroon, which has a dense population attributed to the presence of universities, tourist sites, an oil refinery, and plantations, contributing to increased vehicle traffic and RTI risk. The Limbe Regional Hospital is a 200-bed trauma center and referral hospital with an emergency and casualty department, an outpatient department, and a general surgery unit. Saint Luke Hospital offers orthopedic services to trauma patients, including RTI victims. Buea Regional Hospital is a 120-bed capacity referral hospital with an orthopedic department, surgical unit, emergency unit, outpatient department, and mental health unit for trauma care [24,25]. The study population consisted of all RTI patients presenting to Buea Regional Hospital, Saint Luke Hospital Buea, or Limbe Regional Hospital between January 1^st^, 2019, and December 31^st^, 2023. The locations of the three study hospital sites within Fako Division-Cameroon, with base map produced using QGIS and shape files obtained from the Humanitarian Data Exchange in collaboration with OCHA is shown on Fig 1.

### Inclusion and exclusion criteria

We included records of all RTI patients presenting to these three hospitals within the specified period, with injuries sustained as a result of a road traffic injury (RTI). Records of patients with incomplete data were excluded from the analysis.

### Data collection

Trained data collectors systematically reviewed hospital records, including consultation, hospitalization, and trauma registers, as well as RTI patients’ files, from relevant units involved with RTI care including emergency, surgery, intensive care, orthopedics, and outpatient departments. A data extraction form was developed, pilot-tested at the Solidarity Clinic Buea (a hospital with experience in RTI care), and refined to ensure suitability for the study. The form captured demographic characteristics, injury-related characteristics, hospital care characteristics, PTSD screening recommendation, and PTSD screening uptake. PTSD screening was recommended at least one month after the road crash to allow for the resolution of acute stress responses [26]. PTSD screening (done at ≥ 1 month after RTI) was conducted during hospitalization and in some cases at follow-up visits at the hospital. Data were collected using Kobo Toolbox, a free and open-source suite of tools for field data collection using smartphones and other mobile devices [27]. To ensure that each patient was represented only once in the dataset, we implemented a de-duplication process. Data collectors used unique identifiers (patient name, date of birth, hospital registration number) to identify potential duplicate records. These records were then carefully reviewed to determine if they belonged to the same individual. Discrepancies were resolved by examining the medical records and merging the information into a single, comprehensive record for each patient.

### Data analysis

Data were retrieved from Kobo Toolbox, exported to Microsoft Excel version 13 for initial cleaning, and subsequently imported into SPSS version 25 for statistical analysis. During data cleaning, duplicates were avoided by removing duplicates in Excel and verifying data integrity after removal to ensure no vital information was lost. Age categories were chosen based on groupings in epidemiological studies to allow for comparison. Categorical variables were presented as frequencies and percentages, while continuous variables were presented as means with standard deviations. For classification of the anatomical location of traumatic injury, injuries affecting more than one body part were classified as multiple injuries. Associations between demographic characteristics, injury characteristics, hospital care characteristics, and PTSD screening recommendation and uptake were analyzed using the Chi-square test or Fisher’s exact test (when at least an expected count in a cell was less than 5) [28]. All variables with P < 0.2 were included in a multivariable logistic regression model to identify independent predictors of PTSD screening recommendation and screening uptake. A *p*-value of less than 0.05 was considered statistically significant.

## Results

### Socio-demographic characteristics of participants

A total of 4,218 RTI patients were received and managed at the three hospitals during the study period. The majority of patients, 91.4% (3854/4218), were received at Limbe Regional Hospital. Patients were predominantly male, 69.6% (2937/4218). The age group, 26–35 years recorded the highest proportion of cases, 31.7% (1339/4218). The mean age of patients was 30.34 (±13.51 standard deviation) years. Most of the RTI patients were employed 66.2% (2792/4218) (Table 1).

**Table 1:** Socio-demographic characteristics of RTI patients, Fako Division, 2019-2023 (n = 4218).

Variable	Category	Percentage (n)
Hospital	Buea Regional Hospital	7.6 (321)
	Saint Luke Hospital	1.0 (43)
	Limbe Regional Hospital	91.4 (3854)
Age (years)	<16	10.1 (427)
	16–25	29.1 (1226)
	26–35	31.7 (1339)
	36–45	16.3 (686)
	46–55	7.5 (316)
	56–65	3.7 (158)
	>65	1.6 (66)
Sex	Female	30.4 (1281)
	Male	69.6 (2937)
Employment status	Employed	66.2 (2792)
	Retired	1.6 (67)
	Student	19.4 (820)
	Unemployed	12.8 (539)

### Injury characteristics of study participants

Crashes frequently occurred at night, 41.7% (356/854). Motorcycles were the most common type of vehicle involved, 53.7% (498/928). The highest number of RTIs was recorded in 2022, 23.1% (975/4218). The most used means of transportation for RTI patients to the hospital was 2–3 wheeled vehicles, 56.7% (1451/2558) (Table 2).

**Table 2:** Injury characteristics of RTI patients in the Fako Division, 2019-2023.

Variable	Category	Percentage (n)
Time of road traffic crash (n = 854)	Morning (6:00 am-11:59 am)	26.5 (226)
	Afternoon (12:00 pm-5:59 pm)	31.9 (272)
	Night (6:00 pm-5:59 am)	41.7 (356)
Year of RTI (n = 4218)	2019	14.4 (594)
	2020	22.2 (938)
	2021	20.2 (850)
	2022	23.1 (975)
	2023	20.4 (861)
Type of vehicle involved (n = 928)	Private car	28.3 (263)
	Taxi	12,8 (119)
	Motorcycle	53.7 (498)
	Truck	1.9 (18)
	Bus	2.9 (27)
	Train	0.1 (1)
	Other	0.2 (2)
Means of transportation to the hospital (n = 2558)	On Foot	6.1 (157)
	Ambulance	1.6 (42)
	2-3 Wheeled vehicle	56.7 (1451)
	4 Wheeled vehicle	34.6 (886)
	Police/ Fire fighter	0.8 (20)
	Other	0.1 (2)

*NB: Type of vehicle: 2–3 Wheeled vehicle = Motorcycles/ tricycles, 4 Wheeled vehicle = Taxi/ Private car/ Bus.

**Fig 2:**
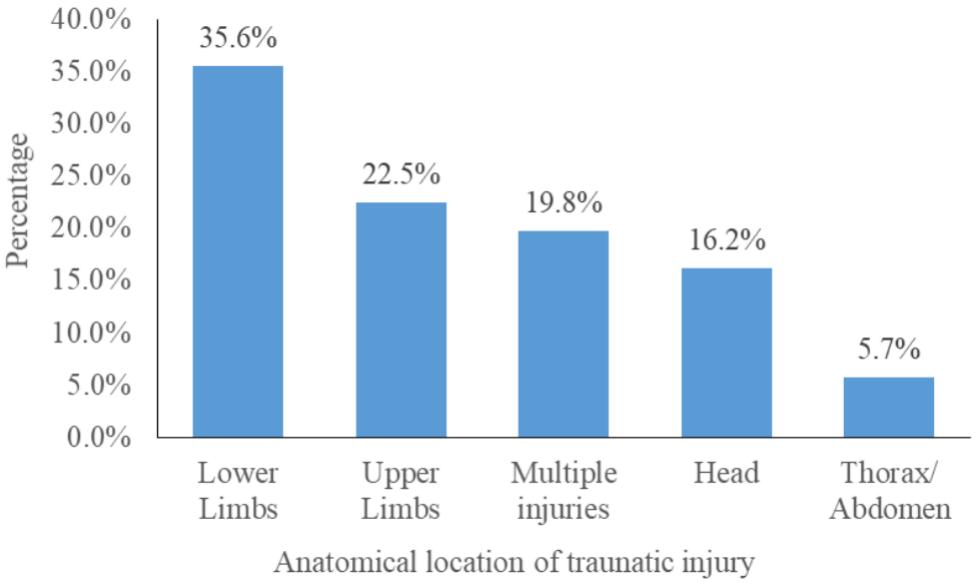
Distribution of RTIs by anatomical location of traumatic injury, Fako, 2019-2023.

Lower limbs, 35.6% (1503/4218), and upper limbs, 22.5% (950/4218), were the most affected anatomical locations for the traumatic injury. Multiple injuries accounted for 19.8% (839/4218) of injuries (Fig 2).

### In-hospital care received and outcomes of road traffic injury patients

Of the RTI patients, 12.7% (534/4218) were hospitalized, and 1.7% (76/4218) were referred (transferred to a higher-level health facility for further treatment or specialized care). Of the hospitalized patients, 93.6% (500/534) received pain medication, 65.2% (348/534) were administered sedatives, and 52.4% (280/534) underwent surgery. Only 11.0% (59/534) of hospitalized patients were recommended for PTSD screening, with an uptake of 44.1% (26/59) among those recommended. Very few hospitalized patients were recommended for psychotherapy, 8.8% (47/534), and an uptake of 85.1% (40/47) among those recommended. In-hospital mortality rate was 5.1% (27/534) (Table 3).

**Table 3:** In-hospital care received and outcomes of RTI patients, Fako, 2019-2023.

Variable	Category	Percentage (n)
Admission status	Not admitted	85.5 (3608)
	Hospitalized	12.7 (534)
	Referred	1.7 (76)
	Total	100 (4218)
Pain medication given	No	6.4 (34)
	Yes	93.6 (500)
	Total	100 (534)
Sedative given	No	34.8 (186)
	Yes	65.2 (348)
	Total	100 (534)
Surgery performed	No	47.6 (254)
	Yes	52.4 (280)
	Total	100 (534)
Screening for PTSD recommended	No	89 (475)
	Yes	11 (59)
	Total	100 (534)
PTSD screening uptake after recommendation	No	55.9 (33)
	Yes	44.1 (26)
	Total	100 (59)
Psychotherapy recommended	No	91.2 (487)
	Yes	8.8 (47)
	Total	100 (534)
Psychotherapy uptake after recommendation	No	14.9 (7)
	Yes	85.1 (40)
	Total	100 (47)
Transfusion performed	No	62.0 (331)
	Yes	38.0 (203)
	Total	100 (534)
Patients’ outcome/ Discharge status after hospitalization	Discharged home	85.4 (456)
	Referred/ transferred to another health facility	3.9 (21)
	Discharged against medical advice	5.6 (30)
	Death	5.1 (27)
	Total	100 (534)

### Association between recommendation for PTSD screening and RTI characteristics

Bivariate analysis revealed significant associations between recommendation for PTSD screening and means of transportation to the hospital (*p* = 0.050), pain medication given (*p* = 0.024), sedative given (*p* <** **0.001), surgery performed (*p* = 0.005), psychotherapy recommended (*p* < 0.001), and transfusion performed (*p* < 0.001). In the multivariable model, sedative given (adjusted odds ratio (AOR) = 6.5, 95%CI = 1.4-30.1, *p* = 0.016), psychotherapy recommended (AOR = 19.2, 95%CI = 8.9-41.6, *p* < 0.000), and transfusion performed (AOR = 3.5, 95%CI = 1.5-7.9, *p* = 0.003) were significantly associated with higher odds for PTSD screening recommendation after controlling for means of transport, pain medication given, and surgery performed (Table 4).

**Table 4:** Association between recommendation for PTSD screening and RTI characteristics.

Variable	Category	PTSD screening recommended Frequency (%)			*P* value	AOR(95%CI)	*P* value
		No (n = 475)	Yes (n = 59)	Total			
Means of transport to the hospital	On Foot	4 (1.7)	4 (6.9)	8 (2.7)			
	Ambulance	15 (6.4)	5 (8.6)	20 (6.8)	0.050		
	2-3 Wheeled vehicle	92 (39.1)	15 (25.9)	107 (36.5)			
	4 Wheeled vehicle	117 (49.8)	34 (58.6)	151 (51.5)			
	Police/ Fire fighter	7 (3.0)	0 (0.0)	7 (2.4)			
	Total	235 (100)	58 (100)	293 (100)			
Pain medication given	No	34 (7.2)	0 (0.0)	34 (6.4)			
	Yes	441 (92.8)	59 (100)	500 (93.6)	0.024		
	Total	475 (100)	59 (100)	534 (100)			
Sedative given	No	184 (38.7)	2 (3.4)	186 (34.8)		Ref	
	Yes	291 (61.3)	57 (96.6)	348 (65.2)	<0.001	6.5 (1.4–30.1)	0.016*
	Total	475 (100)	59 (100)	534 (100)			
Surgery performed	No	236 (49.7)	18 (30.5)	254 (47.6)		Ref	
	Yes	239 (50.3)	41 (69.5)	280 (52.4)	0.005	0.6 (0.3–1.3)	0.175
	Total	475 (100)	59 (100)	534 (100)			
Psychotherapy recommended	No	460 (96.8)	27 (45.8)	487 (91.2)		Ref	
	Yes	15 (3.2)	32 (54.2)	47 (8.8)	<0.001	19.2 (8.9–41.6)	<0.000*
	Total	475 (100)	59 (100)	534 (100)			
Transfusion performed	No	320 (67.4)	11 (18.6)	331 (62.0)		Ref	
	Yes	155 (32.6)	48 (81.4)	203 (38)	<0.001	3.5 (1.5–7.9)	0.003*
	Total	475 (100)	59 (100)	534 (100)			
Discharge status	Discharged home	403 (84.8)	53 (89.8)	456 (85.4)			
	Referred	19 (4.0)	2 (3.4)	21 (3.9)	0.902		
	Discharged against medical advice	28 (5.9)	2 (3.4)	30 (5.6)			
	Death	25 (5.3)	2 (3.4)	27 (5.1)			
	Total	475 (100)	59 (100)	534 (100)			

NB: AOR = adjusted odd ratio, CI = Confidence interval, * = *p*-value<0.05, 2–3 wheeled vehicle = motorcycles/bicycles/tricycles, 4 Wheeled vehicle = Taxi/Private car/Bus.

### Association between PTSD screening uptake after recommendation, and RTI patients’ characteristics

Bivariate analysis revealed a statistically significant association between PTSD screening uptake and recommendation for psychotherapy (*p* = 0.010). Recommendation for psychotherapy was significantly associated with increased PTSD screening uptake (COR = 4.2, 95%CI = 1.4-12.7, *p* = 0.012) (Table 5).

**Table 5:** Association between PTSD screening uptake after recommendation, and demographic, crash-related and in-hospital characteristics.

Variable	Category	PTSD screening uptake Frequency (%)			*P* value	COR (95%CI)	*P* value
		No (n = 33)	Yes (n = 26)	Total			
Age (years)	<16	4 (12.1)	2 (7.7)	6 (10.2)			
	16–25	7 (21.2)	8 (30.8)	15 (25.4)	0.384		
	26–35	7 (21.2)	9 (34.6)	16 (27.1)			
	36–45	11 (33.3)	3 (11.5)	14 (23.7)			
	46–55	1 (3.0)	1 (3.8)	2 (3.4)			
	56–65	2 (6.1)	3 (11.5)	5 (8.5)			
	>65	1 (3.0)	0 (0.0)	1 (1.7)			
	Total	33 (100)	26 (100)	59 (100)			
Psychotherapy recommended	No	20 (60.6)	7 (26.9)	27 (45.8)		Ref	
	Yes	13 (39.4)	19 (73.1)	32 (54.2)	0.010	4.2 (1.4–12.7)	0.012*
	Total	33 (100)	26 (100)	59 (100)			
Discharge status	Discharged home	30 (90.9)	23 (88.5)	53 (89.8)			
	Referred/ transferred to another health facility	1 (3.0)	1 (3.8)	2 (3.4)	1.000		
	Discharged against medical advice	1 (3.0)	1 (3.8)	2 (3.4)			
	Death	1 (3.0)	1 (3.8)	2 (3.4)			
	Total	33 (100)	26 (100)	59 (100)			

NB: COR= Crude odd ratio, CI= Confidence interval, *= *p*-value<0.05.

## Discussion

This study aimed to determine the epidemiological profile of RTI Patients and PTSD screening uptake in hospitals in Fako Division, Cameroon. Our study revealed that few patients were recommended for PTSD screening, and PTSD screening uptake was low, with only 11.0% of hospitalized patients being recommended for PTSD screening, and only 44.1% uptake among those recommended. This underscores significant under-recognition and under-utilization of mental health services.

The demographic characteristics of RTI patients in our study are consistent with previous findings in Cameroon and other settings, with males predominantly affected (69.6%), likely due to their increased mobility, increased likelihood of work outside home, and risk-taking behaviors [8,10,21,29]. This trend is also observed globally, with studies reporting similar male predominance in RTIs [30–35]. The mean age (±standard deviation) of RTI patients was 30.3 (±13.5) years, similar to results reported in Yaounde (31years) [29], and India (32.7 years) [30]. The age groups 16–25 and 26–35 were the most involved in RTIs. These findings are consistent with studies in Rwanda, Iran, and Nepal [36–38]. In contrast, a higher mean age of 41.4 years has been reported in Serbia, probably due to the aging population in Serbia [31]. The fact that the majority of patients were treated at the Limbe Regional Hospital may reflect its status as a major trauma center in the region. While the data from the other two hospitals represent a smaller proportion of the overall sample, we believe that including this data provides a more complete picture of the RTI situation in Fako Division.

Road crashes frequently occurred at night (41.7%), consistent with previous studies [10,30,39]. This finding may be associated with elevated traffic, higher speeds, reduced visibility due to inadequate lighting on roads, roads without traffic panels, poorly marked roads, poorly maintained roads, and driving under the influence. Motorcycles were implicated in over half the RTIs (53.7%), which potentially reflects the high prevalence of commercial motorcycle use and inadequate training among riders. This finding is consistent with previous studies [21,22,40–42]. Ambulance transport was rare (1.6%), similar to 14.6% RTI cases transported by ambulance reported in New Delhi [30] and 3.9% [5] in Cameroon. This highlights gaps in pre-hospital care. Lower (50.6%) and upper limb (38.9%) injuries were most common, mirroring previous studies [23,43,44]. Our findings contrast with those of Rajčević and colleagues in 2024 in Serbia, where head and neck injuries were the most affected parts (37.8%) [31].

Low PTSD screening recommendation (11.0%) and uptake (44.1%) rates highlight limitations in addressing psychological sequelae, despite high PTSD prevalence after RTIs (22.2% globally; 1 in 4 in Africa) [13,14]. General practitioners are likely to recommend PTSD screening for patients who exhibit symptoms of PTSD and/or distress, which clearly shows the psychological impact of RTI on patients and the need for PTSD screening and timely intervention. Subjective screening recommendations, rather than routine protocols, likely contribute to the underestimation of PTSD burden.

Sedative given (AOR = 6.5, 95%CI = 1.4-30.1), psychotherapy recommendation (AOR = 19.2, 95%CI = 8.9-41.6), and transfusion performed (AOR = 3.5, 95%CI = 1.5-7.9) were significant predictors associated with six, nineteen, and three times higher odds, respectively, for PTSD screening recommendation compared to when these services were not offered. These interventions are typically reserved for more critical cases, where the risk of psychological sequelae like PTSD is also higher. This finding underscores the importance of integrating mental health assessments into the care pathway for patients with severe traumatic injuries. These findings suggest that general practitioners are more likely to recommend PTSD screening for patients with severe injuries or those who exhibit distressing symptoms. The significant association between psychotherapy recommendation and PTSD screening recommendation (AOR = 19.2, *p* < 0.000) suggests awareness that formal screening is warranted when psychotherapy is considered beneficial. Our study did not collect data on the specific types of pain medication and sedatives administered to RTI patients. This information was not consistently documented in the medical records and was beyond the scope of our primary research question.

Patients who were recommended for psychotherapy were four times (COR = 4.2(1.4-12.7) more likely to uptake PTSD screening compared to those who were not recommended. This highlights the critical role of clinician endorsement in promoting patient engagement with mental health services, as patients are more likely to pursue screening when a healthcare provider explicitly recommends psychotherapy, suggesting that the provider’s recommendation instills confidence and reduces stigma associated with mental health care. However, low uptake reflects barriers like limited awareness, access, or financial constraints. This underscores the need for training healthcare providers to confidently and routinely recommend screening for PTSD and psychotherapy when appropriate. To improve patient outcomes, timely referrals for PTSD screening and subsequently psychotherapy (a first-line treatment option for PTSD) [45] must be included in routine RTI patients’ care. This will increase the likelihood of PTSD screening uptake and early diagnosis as well as treatment.

Psychotherapy was recommended by general practitioners to some RTI patients who were not recommended for PTSD screening (15 patients from Table 4). This was because some patients had other mental health symptoms leading to a psychotherapy recommendation independent of PTSD screening. However, our data did not include information on patients’ mental health history prior to the road traffic crash. This is a limitation of our study, as pre-existing mental health conditions may influence the recommendation for psychotherapy following RTIs.

### Strengths and limitations

The study’s strengths include a representative sample and multi-hospital data, and the use of real-world clinical data. Additionally, the study focuses on PTSD screening, which is a critical aspect of mental health care for RTI patients. The limitations include a retrospective design subject to data quality issues, which was mitigated by ensuring that only completed data with variables needed for our study were included. Due to poor documentation, it was not always possible to determine the exact timing (though generally recommended ≥1 month post RTI) of the recommendation or the screening in our study.

## Conclusion

Road traffic injuries predominantly affected males and involved motorcycles, with lower and upper limbs being the most frequently injured. The PTSD screening recommendation and uptake among RTI patients in Fako Division were low. The low rates of PTSD screening, coupled with the potential for significant psychological distress following RTIs, underscore a limitation in mental health care for this vulnerable population. The significant association between psychotherapy recommendation and PTSD screening uptake highlights a clear pathway to improved patient engagement: integrating accessible mental health support into routine trauma care. This study highlights the urgent need for integrated trauma care, encompassing routine PTSD screening, timely access to evidence-based psychotherapy, and a strengthened mental health infrastructure. Improving access to mental health care for RTI patients in resource-limited settings like Fako Division is essential for addressing health disparities. This study provides crucial baseline data on PTSD screening rates and associated factors, which can inform the development of targeted interventions to improve mental health service delivery. Further research is needed on actual PTSD burden and screening practices, exploring the barriers and facilitators to PTSD screening and treatment in this specific context.

## Supporting information

S1 Data The original dataset (anonymized). This Excel file contains the raw anonymized data collected for this study, including demographic, injury, and PTSD screening variables.
(XLSX)

S2 Data The anonymized SPSS dataset. This SPSS file contains the anonymized dataset used for statistical analysis, including all variables from S1 Data in SPSS format.
(SAV)

## Abbreviations

RTI: road traffic injury
PTSD: post traumatic stress disorder
RTC: road traffic crash
MH: mental health
